# Ethyl 4-hydr­oxy-2,6-diphenyl-1-(2-thio­morpholinopropano­yl)-1,2,5,6-tetra­hydro­pyridine-3-carboxyl­ate

**DOI:** 10.1107/S1600536809049186

**Published:** 2009-11-21

**Authors:** G. Aridoss, D. Gayathri, Keun Soo Park, Jong Tae Kim, Yeon Tae Jeong

**Affiliations:** a Division of Image Science and Information Engineering, Pukyong National University, Busan 608-739, Republic of Korea; b Institute of Structural Biology and Biophysics-2: Molecular Biophysics, Research Centre Jülich, D-52425 Jülich, Germany

## Abstract

In the title compound, C_27_H_32_N_2_O_4_S, the thio­morpholine ring adopts a chair conformation and the tetra­hydro­pyridine ring is in a distorted envelope conformation. The mol­ecular structure is stabilized by an intra­molecular O—H⋯O inter­action and the crystal packing is stabilized by an inter­molecular C—H⋯O inter­action, generating an *S*(6) motif and a dimer of the type *R*_2_^2^(18), respectively.

## Related literature

For the synthesis and biological activity of 2,6-diaryl­piperidin-4-one derivatives, see: Aridoss, Balasubramanian, Parthiban, Ramachandran & Kabilan (2007 ▶); Aridoss, Balasubramanian, Parthiban & Kabilan (2007 ▶); Aridoss, Parthiban *et al.* (2009 ▶). For a related structure, see: Aridoss, Gayathri *et al.* (2009 ▶). For ring conformational analysis, see: Cremer & Pople (1975 ▶); Nardelli (1983 ▶).
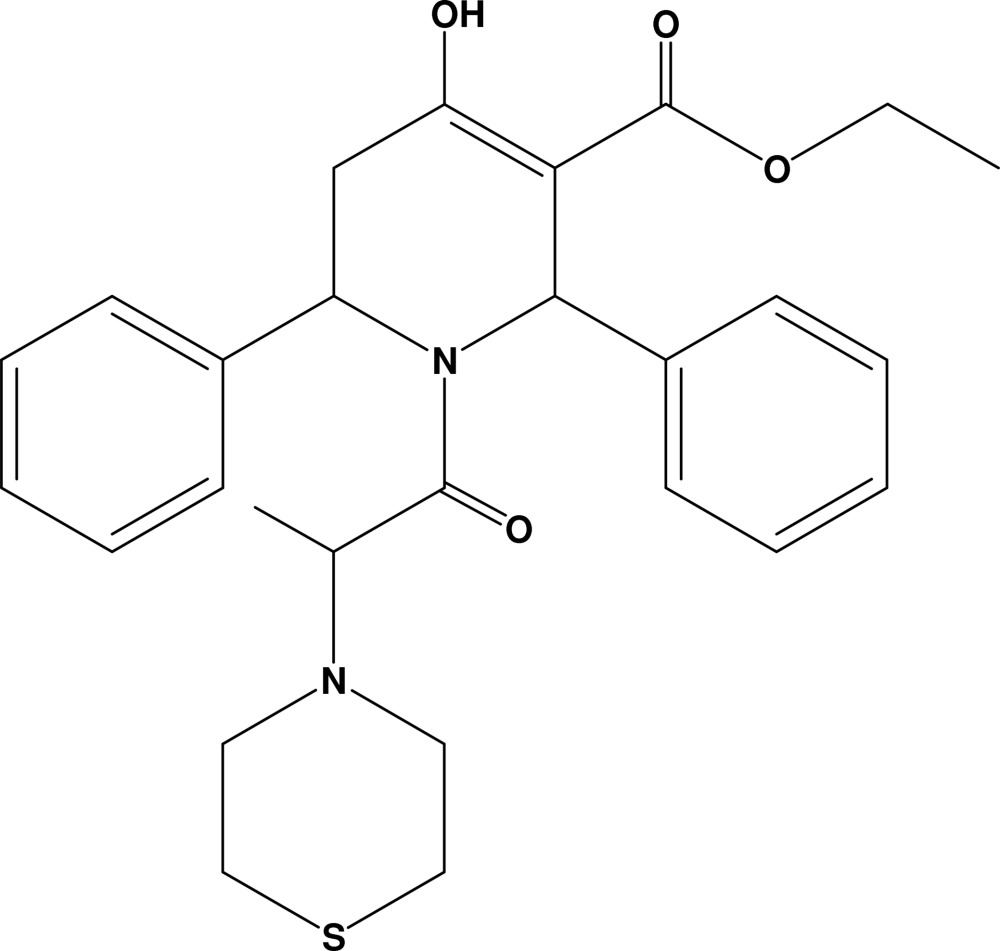


## Experimental

### 

#### Crystal data

C_27_H_32_N_2_O_4_S
*M* *_r_* = 480.61
Triclinic, 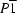

*a* = 9.904 (3) Å
*b* = 11.400 (4) Å
*c* = 12.103 (4) Å
α = 93.908 (18)°
β = 104.941 (15)°
γ = 106.819 (16)°
*V* = 1248.7 (7) Å^3^
*Z* = 2
Mo *K*α radiation
μ = 0.17 mm^−1^
*T* = 293 K
0.30 × 0.25 × 0.20 mm

#### Data collection

Bruker Kappa APEXII CCD diffractometer
Absorption correction: multi-scan (**SADABS**; Bruker, 1999 ▶) *T*_min_ = 0.952, *T*_max_ = 0.968
26182 measured reflections
5707 independent reflections
4543 reflections with *I* > 2σ(*I*)
*R*_int_ = 0.032

#### Refinement

*R*[*F* ^2^ > 2σ(*F* ^2^)] = 0.038
*wR*(*F* ^2^) = 0.117
*S* = 1.01
5707 reflections
309 parameters
H-atom parameters constrained
Δρ_max_ = 0.21 e Å^−3^
Δρ_min_ = −0.36 e Å^−3^

### 

Data collection: *APEX2* (Bruker, 2004 ▶); cell refinement: *SAINT* (Bruker, 2004 ▶); data reduction: *SAINT*; program(s) used to solve structure: *SIR92* (Altomare *et al.*, 1994 ▶); program(s) used to refine structure: *SHELXL97* (Sheldrick, 2008 ▶); molecular graphics: *PLATON* (Spek, 2009 ▶); software used to prepare material for publication: *SHELXL97*.

## Supplementary Material

Crystal structure: contains datablocks I, global. DOI: 10.1107/S1600536809049186/is2490sup1.cif

Structure factors: contains datablocks I. DOI: 10.1107/S1600536809049186/is2490Isup2.hkl

Additional supplementary materials:  crystallographic information; 3D view; checkCIF report

## Figures and Tables

**Table 1:** Hydrogen-bond geometry (Å, °)

*D*—H⋯*A*	*D*—H	H⋯*A*	*D*⋯*A*	*D*—H⋯*A*
O2—H2⋯O3	0.82	1.85	2.560 (2)	144
C24—H24*B*⋯O3^i^	0.96	2.54	3.285 (2)	135

Symmetry code: (i) 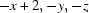.

**Fig. 1.**
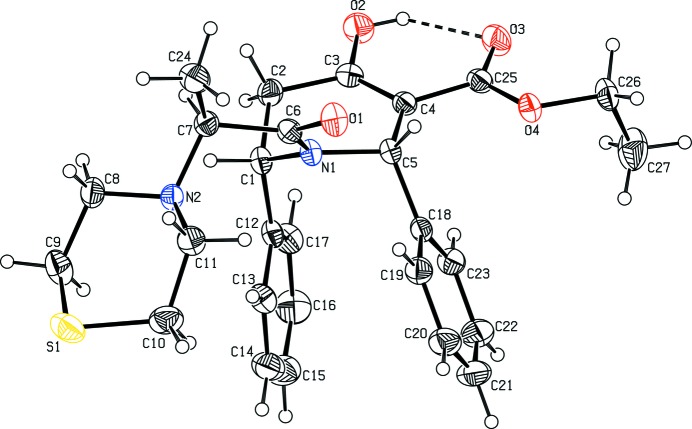
The molecular structure of title compound, showing 30% probability displacement ellipsoids.

**Fig. 2.**
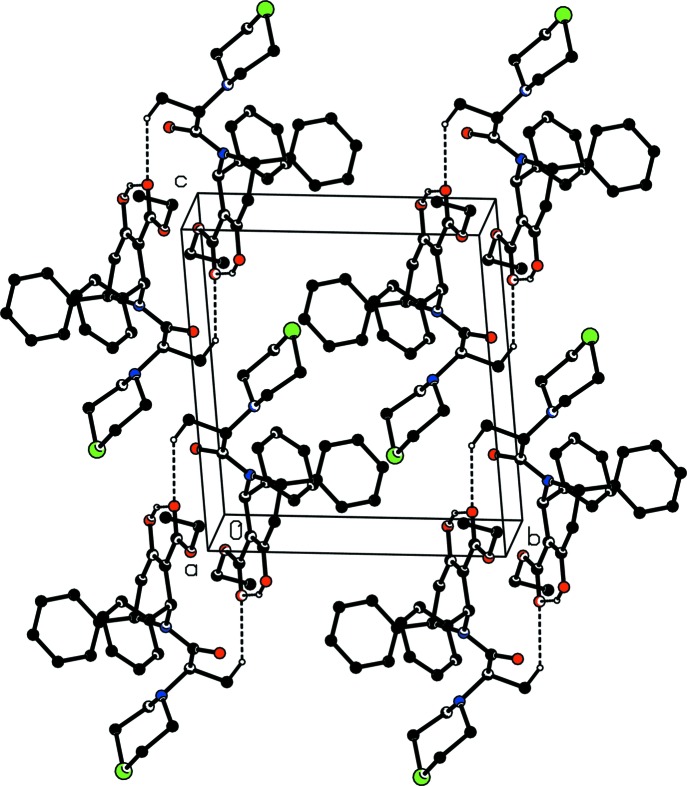
The molecular packing of (I). For clarity, hydrogen atoms which are not involved in hydrogen bonding are omitted.

## supplementary crystallographic information

### Comment

Our current research work is committed to find 2,6-diarylpiperidin-4-one based lead drug for the antimicrobial therapy and exploring the stereochemistry of its *N*-acyl derivatives (Aridoss, Balasubramanian, Parthiban, Ramachandran & Kabilan, 2007; Aridoss, Balasubramanian, Parthiban & Kabilan, 2007; Aridoss, Parthiban *et al.*, 2009). Recently we have disclosed the crystal structure of ethyl 1-(2-bromopropanoyl)-4-hydroxy-2,6-diphenyl-1,2,5,6- tetrahydropyridin-3-carboxylate (Aridoss, Gayathri *et al.*, 2009), which crystallizes with two independent molecules per asymmetric unit. Here, the tetrahydropyridine ring adopts a half-chair conformation in one molecule and distorted envelope conformation in other molecule. Thus to understand the change in conformation of the above said compound upon nucleophilic substitution of thiomorpholine in place of bromine, crystal structure of the title compound is determined by X-ray diffraction study and discussed in this paper.

The sum of the angles at N1 [358.6 (3)°] and N2 [336.1 (3)°] are in accordance with *sp*^2^ and *sp*^3^ hybridization, respectively. The dihedral angle between the two phenyl rings attached to the pyridine moiety is 21.8 (1)°. The thiomorpholine ring adopts chair conformation with atoms C9 and C11 deviating by 0.758 (2) and -0.673 (2) Å, respectively, from the least squares plane defined by atoms N2/C8/S1/C10. The tetrahydropyridine ring adops distorted envelope conformation. The puckering parameters (Cremer & Pople, 1975) and the smallest displacement asymmetry parameters (Nardelli, 1983) for thiomorpholine and tetrahydropyridine rings are q_2_ = 0.065 (1), 0.394 (1) Å, q_3_ = 0.639 (2), 0.294 (1) Å; Q_T_ = 0.642 (2), 0.491 (1) Å and θ = 5.7 (1), 53.3 (2)°, respectively.

The molecular structure and the crystal packing are stabilized by O—H···O intramolecular and C—H···O intermolecular interactions, respectively, with atom O3 acting as bifurcated acceptor. In intramolecular interaction, atom O2 acts as a donor to O3 generating an S(6) motif and in intermolecular interaction, atom C24 acts as a donor to atom O3 at (2 - *x*, -*y*, -*z*), generating a dimer of the type *R*_2_^2^(18).

### Experimental

To a solution of thiomorpholine (1 equiv.) and dry K_2_CO_3_ in benzene, ethyl 1-(2-bromopropanoyl)-4-hydroxy-2,6-diphenyl-1,2,5,6-tetrahydropyridin-3- carboxylate (1 equiv.; Aridoss, Gayathri *et al.*, 2009) in benzene was added slowly over a period of 15 minutes and refluxed over night. After the completion of reaction, the contents were poured into water and extracted twice with ethyl acetate. The combined organic extracts were then washed well with brine and dried over anhydrous sodium sulfate. This upon evaporation, column purification and subsequent recrystallization in distilled ethanol afforded fine white crystals appropriate for X-ray diffraction study.

### Refinement

All H atoms were refined using a riding model, with C—H = 0.93 Å and *U*_iso_(H) = 1.2*U*_eq_(C) for aromatic, C—H = 0.98 Å and *U*_iso_(H) = 1.2*U*_eq_(C) for CH, C—H = 0.97 Å and *U*_iso_(H) = 1.2*U*_eq_(C) for CH_2_, C—H = 0.96 Å and *U*_iso_(H) = 1.5*U*_eq_(C) for CH_3_, and O—H = 0.82 Å and *U*_iso_(H) = 1.5*U*_eq_(O) for the OH group.

### Crystal data

**Table:**

C_27_H_32_N_2_O_4_S	*Z* = 2
*M**_r_* = 480.61	*F*(000) = 512
Triclinic, *P*1	*D*_x_ = 1.278 Mg m^−^^3^
Hall symbol: -P 1	Mo *K*α radiation, λ = 0.71073 Å
*a* = 9.904 (3) Å	Cell parameters from 5338 reflections
*b* = 11.400 (4) Å	θ = 2.3–27.8°
*c* = 12.103 (4) Å	µ = 0.17 mm^−^^1^
α = 93.908 (18)°	*T* = 293 K
β = 104.941 (15)°	Block, colorless
γ = 106.819 (16)°	0.30 × 0.25 × 0.20 mm
*V* = 1248.7 (7) Å^3^	

### Data collection

**Table:**

Bruker Kappa APEXII CCD diffractometer	5707 independent reflections
Radiation source: fine-focus sealed tube	4543 reflections with *I* > 2σ(*I*)
graphite	*R*_int_ = 0.032
ω and φ scan	θ_max_ = 27.8°, θ_min_ = 2.3°
Absorption correction: multi-scan (*SADABS*; Bruker, 1999)	*h* = −12→12
*T*_min_ = 0.952, *T*_max_ = 0.968	*k* = −14→14
26182 measured reflections	*l* = −15→15

### Refinement

**Table:**

Refinement on *F*^2^	Primary atom site location: structure-invariant direct methods
Least-squares matrix: full	Secondary atom site location: difference Fourier map
*R*[*F*^2^ > 2σ(*F*^2^)] = 0.038	Hydrogen site location: inferred from neighbouring sites
*wR*(*F*^2^) = 0.117	H-atom parameters constrained
*S* = 1.00	*w* = 1/[σ^2^(*F*_o_^2^) + (0.0614*P*)^2^ + 0.2715*P*] where *P* = (*F*_o_^2^ + 2*F*_c_^2^)/3
5707 reflections	(Δ/σ)_max_ = 0.001
309 parameters	Δρ_max_ = 0.21 e Å^−^^3^
0 restraints	Δρ_min_ = −0.36 e Å^−^^3^

### Special details

**Table:**

Geometry. All e.s.d.'s (except the e.s.d. in the dihedral angle between two l.s. planes) are estimated using the full covariance matrix. The cell e.s.d.'s are taken into account individually in the estimation of e.s.d.'s in distances, angles and torsion angles; correlations between e.s.d.'s in cell parameters are only used when they are defined by crystal symmetry. An approximate (isotropic) treatment of cell e.s.d.'s is used for estimating e.s.d.'s involving l.s. planes.
Refinement. Refinement of *F*^2^ against ALL reflections. The weighted *R*-factor *wR* and goodness of fit *S* are based on *F*^2^, conventional *R*-factors *R* are based on *F*, with *F* set to zero for negative *F*^2^. The threshold expression of *F*^2^ > σ(*F*^2^) is used only for calculating *R*-factors(gt) *etc*. and is not relevant to the choice of reflections for refinement. *R*-factors based on *F*^2^ are statistically about twice as large as those based on *F*, and *R*- factors based on ALL data will be even larger.

### Fractional atomic coordinates and isotropic or equivalent isotropic displacement parameters (Å^2^)

**Table:**

	*x*	*y*	*z*	*U*_iso_*/*U*_eq_	
C1	1.19148 (14)	0.27730 (12)	0.23357 (11)	0.0347 (3)	
H1	1.2819	0.2883	0.2959	0.042*	
C2	1.22176 (15)	0.24683 (13)	0.12043 (12)	0.0400 (3)	
H2A	1.2817	0.3218	0.1013	0.048*	
H2B	1.2769	0.1885	0.1293	0.048*	
C3	1.08292 (15)	0.19255 (12)	0.02448 (11)	0.0371 (3)	
C4	0.95026 (14)	0.14479 (11)	0.04268 (10)	0.0331 (3)	
C5	0.93213 (13)	0.13505 (11)	0.16238 (10)	0.0310 (3)	
H5	0.8828	0.0472	0.1626	0.037*	
C6	1.10345 (14)	0.08311 (12)	0.31764 (10)	0.0337 (3)	
C7	1.25989 (15)	0.11016 (13)	0.39709 (11)	0.0357 (3)	
H7	1.3274	0.1358	0.3501	0.043*	
C8	1.45086 (15)	0.27218 (15)	0.54032 (13)	0.0478 (4)	
H8A	1.4994	0.2871	0.4801	0.057*	
H8B	1.4889	0.2154	0.5853	0.057*	
C9	1.4863 (2)	0.39292 (17)	0.61776 (16)	0.0611 (4)	
H9A	1.4450	0.4485	0.5733	0.073*	
H9B	1.5924	0.4315	0.6454	0.073*	
C10	1.2279 (2)	0.29267 (17)	0.65584 (14)	0.0574 (4)	
H10A	1.1697	0.2655	0.7082	0.069*	
H10B	1.1886	0.3499	0.6126	0.069*	
C11	1.21338 (17)	0.18191 (15)	0.57289 (12)	0.0457 (3)	
H11A	1.2508	0.1239	0.6163	0.055*	
H11B	1.1099	0.1405	0.5329	0.055*	
C12	1.15095 (14)	0.39523 (12)	0.24545 (11)	0.0358 (3)	
C13	1.12638 (16)	0.43177 (14)	0.34780 (13)	0.0448 (3)	
H13	1.1291	0.3814	0.4051	0.054*	
C14	1.0980 (2)	0.54136 (16)	0.36610 (16)	0.0577 (4)	
H14	1.0809	0.5643	0.4352	0.069*	
C15	1.0948 (2)	0.61727 (16)	0.28258 (18)	0.0640 (5)	
H15	1.0773	0.6922	0.2955	0.077*	
C16	1.1173 (2)	0.58217 (16)	0.18069 (17)	0.0608 (4)	
H16	1.1135	0.6327	0.1235	0.073*	
C17	1.14591 (18)	0.47184 (13)	0.16176 (13)	0.0471 (3)	
H17	1.1618	0.4490	0.0921	0.057*	
C18	0.83851 (13)	0.20571 (12)	0.19968 (11)	0.0332 (3)	
C19	0.80193 (16)	0.18358 (14)	0.30119 (12)	0.0425 (3)	
H19	0.8339	0.1262	0.3429	0.051*	
C20	0.71920 (19)	0.24515 (16)	0.34109 (15)	0.0547 (4)	
H20	0.6955	0.2294	0.4093	0.066*	
C21	0.6719 (2)	0.32964 (16)	0.28039 (16)	0.0597 (4)	
H21	0.6165	0.3720	0.3075	0.072*	
C22	0.7062 (2)	0.35187 (16)	0.17939 (16)	0.0555 (4)	
H22	0.6733	0.4090	0.1379	0.067*	
C23	0.78906 (16)	0.29013 (13)	0.13889 (12)	0.0423 (3)	
H23	0.8116	0.3057	0.0702	0.051*	
C24	1.2782 (2)	−0.00907 (15)	0.43919 (14)	0.0524 (4)	
H24A	1.3719	0.0096	0.4965	0.079*	
H24B	1.2733	−0.0661	0.3751	0.079*	
H24C	1.2008	−0.0457	0.4725	0.079*	
C25	0.82265 (15)	0.08996 (12)	−0.05693 (11)	0.0369 (3)	
C26	0.56361 (17)	−0.00482 (15)	−0.12584 (13)	0.0494 (4)	
H26A	0.4891	−0.0668	−0.1038	0.059*	
H26B	0.5820	−0.0430	−0.1922	0.059*	
C27	0.5094 (2)	0.1005 (2)	−0.15733 (19)	0.0757 (6)	
H27A	0.4967	0.1411	−0.0904	0.114*	
H27B	0.4167	0.0701	−0.2167	0.114*	
H27C	0.5797	0.1585	−0.1852	0.114*	
N1	1.07728 (11)	0.16791 (10)	0.24818 (9)	0.0326 (2)	
N2	1.29325 (11)	0.21563 (10)	0.48737 (9)	0.0336 (2)	
O1	1.00568 (11)	−0.01107 (9)	0.31843 (9)	0.0454 (2)	
O2	1.10662 (12)	0.19398 (11)	−0.07897 (9)	0.0520 (3)	
H2	1.0283	0.1614	−0.1296	0.078*	
O3	0.82742 (12)	0.08937 (11)	−0.15678 (8)	0.0539 (3)	
O4	0.69839 (10)	0.03850 (9)	−0.03050 (8)	0.0406 (2)	
S1	1.41530 (6)	0.37181 (4)	0.73873 (4)	0.06261 (15)	

### Atomic displacement parameters (Å^2^)

**Table:**

	*U*^11^	*U*^22^	*U*^33^	*U*^12^	*U*^13^	*U*^23^
C1	0.0296 (6)	0.0326 (6)	0.0350 (6)	0.0045 (5)	0.0035 (5)	0.0060 (5)
C2	0.0337 (7)	0.0390 (7)	0.0460 (8)	0.0076 (5)	0.0137 (6)	0.0064 (6)
C3	0.0431 (7)	0.0354 (7)	0.0351 (7)	0.0139 (6)	0.0140 (6)	0.0047 (5)
C4	0.0358 (6)	0.0322 (6)	0.0299 (6)	0.0116 (5)	0.0070 (5)	0.0025 (5)
C5	0.0288 (6)	0.0298 (6)	0.0289 (6)	0.0052 (5)	0.0041 (5)	0.0032 (5)
C6	0.0370 (7)	0.0346 (7)	0.0286 (6)	0.0105 (5)	0.0090 (5)	0.0055 (5)
C7	0.0362 (7)	0.0409 (7)	0.0306 (6)	0.0146 (5)	0.0078 (5)	0.0069 (5)
C8	0.0320 (7)	0.0619 (10)	0.0434 (8)	0.0100 (7)	0.0079 (6)	0.0019 (7)
C9	0.0495 (9)	0.0547 (10)	0.0591 (10)	−0.0010 (8)	0.0049 (8)	−0.0027 (8)
C10	0.0655 (11)	0.0708 (11)	0.0464 (9)	0.0303 (9)	0.0250 (8)	0.0069 (8)
C11	0.0438 (8)	0.0530 (9)	0.0390 (7)	0.0094 (7)	0.0169 (6)	0.0063 (6)
C12	0.0307 (6)	0.0302 (6)	0.0368 (7)	0.0014 (5)	0.0032 (5)	0.0031 (5)
C13	0.0459 (8)	0.0404 (7)	0.0407 (7)	0.0057 (6)	0.0097 (6)	0.0036 (6)
C14	0.0615 (10)	0.0485 (9)	0.0599 (10)	0.0124 (8)	0.0215 (8)	−0.0046 (8)
C15	0.0691 (12)	0.0387 (9)	0.0863 (13)	0.0184 (8)	0.0259 (10)	0.0066 (8)
C16	0.0725 (11)	0.0420 (9)	0.0709 (11)	0.0196 (8)	0.0213 (9)	0.0216 (8)
C17	0.0546 (9)	0.0389 (8)	0.0446 (8)	0.0112 (7)	0.0125 (7)	0.0101 (6)
C18	0.0293 (6)	0.0327 (6)	0.0313 (6)	0.0049 (5)	0.0051 (5)	0.0003 (5)
C19	0.0429 (7)	0.0467 (8)	0.0379 (7)	0.0131 (6)	0.0130 (6)	0.0062 (6)
C20	0.0576 (10)	0.0613 (10)	0.0484 (9)	0.0173 (8)	0.0248 (8)	0.0013 (7)
C21	0.0619 (10)	0.0562 (10)	0.0693 (11)	0.0254 (8)	0.0285 (9)	−0.0019 (8)
C22	0.0611 (10)	0.0474 (9)	0.0641 (10)	0.0279 (8)	0.0167 (8)	0.0086 (8)
C23	0.0455 (8)	0.0413 (7)	0.0403 (7)	0.0150 (6)	0.0117 (6)	0.0060 (6)
C24	0.0642 (10)	0.0485 (9)	0.0429 (8)	0.0280 (8)	0.0018 (7)	0.0064 (7)
C25	0.0422 (7)	0.0358 (7)	0.0311 (6)	0.0146 (6)	0.0065 (5)	0.0017 (5)
C26	0.0395 (8)	0.0513 (9)	0.0411 (8)	0.0069 (6)	−0.0052 (6)	−0.0004 (6)
C27	0.0595 (11)	0.0746 (13)	0.0794 (13)	0.0234 (10)	−0.0081 (10)	0.0230 (11)
N1	0.0296 (5)	0.0308 (5)	0.0314 (5)	0.0059 (4)	0.0030 (4)	0.0054 (4)
N2	0.0291 (5)	0.0398 (6)	0.0302 (5)	0.0091 (4)	0.0077 (4)	0.0055 (4)
O1	0.0431 (5)	0.0415 (5)	0.0446 (5)	0.0043 (4)	0.0088 (4)	0.0161 (4)
O2	0.0516 (6)	0.0649 (7)	0.0388 (5)	0.0120 (5)	0.0204 (5)	0.0043 (5)
O3	0.0537 (6)	0.0723 (8)	0.0297 (5)	0.0162 (6)	0.0084 (4)	0.0000 (5)
O4	0.0352 (5)	0.0447 (5)	0.0327 (5)	0.0077 (4)	0.0010 (4)	0.0018 (4)
S1	0.0835 (3)	0.0571 (3)	0.0407 (2)	0.0286 (2)	0.0031 (2)	−0.00484 (18)

### Geometric parameters (Å, °)

**Table:**

C1—N1	1.4773 (16)	C12—C13	1.382 (2)
C1—C2	1.5142 (19)	C13—C14	1.373 (2)
C1—C12	1.5182 (19)	C13—H13	0.9300
C1—H1	0.9800	C14—C15	1.375 (3)
C2—C3	1.4861 (19)	C14—H14	0.9300
C2—H2A	0.9700	C15—C16	1.363 (3)
C2—H2B	0.9700	C15—H15	0.9300
C3—O2	1.3323 (16)	C16—C17	1.384 (2)
C3—C4	1.3501 (19)	C16—H16	0.9300
C4—C25	1.4477 (18)	C17—H17	0.9300
C4—C5	1.5126 (17)	C18—C23	1.375 (2)
C5—N1	1.4665 (16)	C18—C19	1.3867 (19)
C5—C18	1.5202 (18)	C19—C20	1.374 (2)
C5—H5	0.9800	C19—H19	0.9300
C6—O1	1.2227 (16)	C20—C21	1.367 (3)
C6—N1	1.3609 (17)	C20—H20	0.9300
C6—C7	1.5281 (18)	C21—C22	1.371 (2)
C7—N2	1.4675 (18)	C21—H21	0.9300
C7—C24	1.526 (2)	C22—C23	1.380 (2)
C7—H7	0.9800	C22—H22	0.9300
C8—N2	1.4520 (18)	C23—H23	0.9300
C8—C9	1.507 (2)	C24—H24A	0.9600
C8—H8A	0.9700	C24—H24B	0.9600
C8—H8B	0.9700	C24—H24C	0.9600
C9—S1	1.782 (2)	C25—O3	1.2212 (17)
C9—H9A	0.9700	C25—O4	1.3310 (17)
C9—H9B	0.9700	C26—O4	1.4505 (16)
C10—C11	1.507 (2)	C26—C27	1.485 (3)
C10—S1	1.792 (2)	C26—H26A	0.9700
C10—H10A	0.9700	C26—H26B	0.9700
C10—H10B	0.9700	C27—H27A	0.9600
C11—N2	1.4647 (17)	C27—H27B	0.9600
C11—H11A	0.9700	C27—H27C	0.9600
C11—H11B	0.9700	O2—H2	0.8200
C12—C17	1.382 (2)		
			
N1—C1—C2	107.66 (11)	C12—C13—H13	119.5
N1—C1—C12	112.16 (11)	C13—C14—C15	120.20 (16)
C2—C1—C12	114.82 (11)	C13—C14—H14	119.9
N1—C1—H1	107.3	C15—C14—H14	119.9
C2—C1—H1	107.3	C16—C15—C14	119.63 (16)
C12—C1—H1	107.3	C16—C15—H15	120.2
C3—C2—C1	111.58 (11)	C14—C15—H15	120.2
C3—C2—H2A	109.3	C15—C16—C17	120.40 (16)
C1—C2—H2A	109.3	C15—C16—H16	119.8
C3—C2—H2B	109.3	C17—C16—H16	119.8
C1—C2—H2B	109.3	C12—C17—C16	120.56 (15)
H2A—C2—H2B	108.0	C12—C17—H17	119.7
O2—C3—C4	125.09 (13)	C16—C17—H17	119.7
O2—C3—C2	112.09 (12)	C23—C18—C19	118.54 (13)
C4—C3—C2	122.79 (12)	C23—C18—C5	123.75 (12)
C3—C4—C25	118.29 (12)	C19—C18—C5	117.71 (12)
C3—C4—C5	122.64 (11)	C20—C19—C18	120.98 (14)
C25—C4—C5	118.79 (11)	C20—C19—H19	119.5
N1—C5—C4	109.82 (10)	C18—C19—H19	119.5
N1—C5—C18	110.60 (10)	C21—C20—C19	119.88 (15)
C4—C5—C18	116.73 (10)	C21—C20—H20	120.1
N1—C5—H5	106.3	C19—C20—H20	120.1
C4—C5—H5	106.3	C20—C21—C22	119.83 (15)
C18—C5—H5	106.3	C20—C21—H21	120.1
O1—C6—N1	121.77 (12)	C22—C21—H21	120.1
O1—C6—C7	120.49 (12)	C21—C22—C23	120.49 (15)
N1—C6—C7	117.73 (11)	C21—C22—H22	119.8
N2—C7—C24	116.11 (11)	C23—C22—H22	119.8
N2—C7—C6	109.40 (10)	C18—C23—C22	120.26 (14)
C24—C7—C6	109.39 (12)	C18—C23—H23	119.9
N2—C7—H7	107.2	C22—C23—H23	119.9
C24—C7—H7	107.2	C7—C24—H24A	109.5
C6—C7—H7	107.2	C7—C24—H24B	109.5
N2—C8—C9	111.80 (13)	H24A—C24—H24B	109.5
N2—C8—H8A	109.3	C7—C24—H24C	109.5
C9—C8—H8A	109.3	H24A—C24—H24C	109.5
N2—C8—H8B	109.3	H24B—C24—H24C	109.5
C9—C8—H8B	109.3	O3—C25—O4	122.39 (12)
H8A—C8—H8B	107.9	O3—C25—C4	123.53 (13)
C8—C9—S1	112.18 (12)	O4—C25—C4	114.08 (11)
C8—C9—H9A	109.2	O4—C26—C27	110.13 (13)
S1—C9—H9A	109.2	O4—C26—H26A	109.6
C8—C9—H9B	109.2	C27—C26—H26A	109.6
S1—C9—H9B	109.2	O4—C26—H26B	109.6
H9A—C9—H9B	107.9	C27—C26—H26B	109.6
C11—C10—S1	112.47 (12)	H26A—C26—H26B	108.1
C11—C10—H10A	109.1	C26—C27—H27A	109.5
S1—C10—H10A	109.1	C26—C27—H27B	109.5
C11—C10—H10B	109.1	H27A—C27—H27B	109.5
S1—C10—H10B	109.1	C26—C27—H27C	109.5
H10A—C10—H10B	107.8	H27A—C27—H27C	109.5
N2—C11—C10	112.61 (13)	H27B—C27—H27C	109.5
N2—C11—H11A	109.1	C6—N1—C5	117.56 (10)
C10—C11—H11A	109.1	C6—N1—C1	125.05 (11)
N2—C11—H11B	109.1	C5—N1—C1	115.92 (10)
C10—C11—H11B	109.1	C8—N2—C11	112.46 (11)
H11A—C11—H11B	107.8	C8—N2—C7	111.81 (11)
C17—C12—C13	118.22 (14)	C11—N2—C7	111.79 (11)
C17—C12—C1	123.25 (13)	C3—O2—H2	109.5
C13—C12—C1	118.41 (12)	C25—O4—C26	116.77 (11)
C14—C13—C12	120.97 (15)	C9—S1—C10	95.98 (8)
C14—C13—H13	119.5		
			
N1—C1—C2—C3	48.04 (14)	C18—C19—C20—C21	0.0 (2)
C12—C1—C2—C3	−77.64 (14)	C19—C20—C21—C22	0.5 (3)
C1—C2—C3—O2	164.35 (11)	C20—C21—C22—C23	−0.4 (3)
C1—C2—C3—C4	−17.41 (18)	C19—C18—C23—C22	0.6 (2)
O2—C3—C4—C25	0.3 (2)	C5—C18—C23—C22	−179.08 (13)
C2—C3—C4—C25	−177.71 (12)	C21—C22—C23—C18	−0.2 (2)
O2—C3—C4—C5	174.15 (12)	C3—C4—C25—O3	−3.3 (2)
C2—C3—C4—C5	−3.9 (2)	C5—C4—C25—O3	−177.40 (13)
C3—C4—C5—N1	−7.76 (17)	C3—C4—C25—O4	176.05 (12)
C25—C4—C5—N1	166.06 (11)	C5—C4—C25—O4	1.95 (17)
C3—C4—C5—C18	119.13 (14)	O1—C6—N1—C5	−8.07 (18)
C25—C4—C5—C18	−67.04 (15)	C7—C6—N1—C5	172.90 (10)
O1—C6—C7—N2	−110.36 (14)	O1—C6—N1—C1	−173.66 (12)
N1—C6—C7—N2	68.68 (14)	C7—C6—N1—C1	7.31 (18)
O1—C6—C7—C24	17.86 (18)	C4—C5—N1—C6	−123.91 (12)
N1—C6—C7—C24	−163.10 (12)	C18—C5—N1—C6	105.82 (13)
N2—C8—C9—S1	64.32 (17)	C4—C5—N1—C1	42.99 (14)
S1—C10—C11—N2	−61.25 (16)	C18—C5—N1—C1	−87.27 (13)
N1—C1—C12—C17	−125.18 (14)	C2—C1—N1—C6	101.21 (14)
C2—C1—C12—C17	−1.86 (18)	C12—C1—N1—C6	−131.53 (13)
N1—C1—C12—C13	58.79 (15)	C2—C1—N1—C5	−64.58 (14)
C2—C1—C12—C13	−177.89 (12)	C12—C1—N1—C5	62.67 (14)
C17—C12—C13—C14	−0.1 (2)	C9—C8—N2—C11	−63.15 (17)
C1—C12—C13—C14	176.10 (14)	C9—C8—N2—C7	170.12 (13)
C12—C13—C14—C15	−0.5 (3)	C10—C11—N2—C8	61.90 (17)
C13—C14—C15—C16	1.1 (3)	C10—C11—N2—C7	−171.36 (12)
C14—C15—C16—C17	−1.1 (3)	C24—C7—N2—C8	75.03 (16)
C13—C12—C17—C16	0.2 (2)	C6—C7—N2—C8	−160.59 (11)
C1—C12—C17—C16	−175.88 (14)	C24—C7—N2—C11	−52.06 (16)
C15—C16—C17—C12	0.5 (3)	C6—C7—N2—C11	72.31 (14)
N1—C5—C18—C23	116.73 (13)	O3—C25—O4—C26	−7.80 (19)
C4—C5—C18—C23	−9.78 (18)	C4—C25—O4—C26	172.84 (11)
N1—C5—C18—C19	−62.99 (15)	C27—C26—O4—C25	−81.20 (18)
C4—C5—C18—C19	170.50 (11)	C8—C9—S1—C10	−55.24 (14)
C23—C18—C19—C20	−0.6 (2)	C11—C10—S1—C9	53.83 (14)
C5—C18—C19—C20	179.18 (13)		

### Hydrogen-bond geometry (Å, °)

**Table:**

*D*—H···*A*	*D*—H	H···*A*	*D*···*A*	*D*—H···*A*
O2—H2···O3	0.82	1.85	2.560 (2)	144
C24—H24B···O3^i^	0.96	2.54	3.285 (2)	135

Symmetry codes: (i) −*x*+2, −*y*, −*z*.

## References

[bb1] Altomare, A., Cascarano, G., Giacovazzo, C., Guagliardi, A., Burla, M. C., Polidori, G. & Camalli, M. (1994). *J. Appl. Cryst.* **27**, 435.

[bb2] Aridoss, G., Balasubramanian, S., Parthiban, P. & Kabilan, S. (2007). *Eur. J. Med. Chem.* **42**, 851–860. 10.1016/j.ejmech.2006.12.005 17275965

[bb3] Aridoss, G., Balasubramanian, S., Parthiban, P., Ramachandran, R. & Kabilan, S. (2007). *Med. Chem. Res.* **16**, 188–204.

[bb4] Aridoss, G., Gayathri, D., Velmurugan, D., Kim, M. S. & Jeong, Y. T. (2009). *Acta Cryst.* E**65**, o1708–o1709. 10.1107/S1600536809023836 PMC2969390 21582960

[bb5] Aridoss, G., Parthiban, P., Ramachandran, R., Prakash, M., Kabilan, S. & Jeong, Y. T. (2009). *Eur. J. Med. Chem.* **44**, 577–592. 10.1016/j.ejmech.2008.03.031 18485539

[bb6] Bruker (1999). *SADABS*. Bruker AXS Inc., Madison, Wisconsin, USA.

[bb7] Bruker (2004). *APEX2* and *SAINT*. Bruker AXS Inc., Madison, Wisconsin, USA.

[bb8] Cremer, D. & Pople, J. A. (1975). *J. Am. Chem. Soc.* **97**, 1354–1358.

[bb9] Nardelli, M. (1983). *Acta Cryst.* C**39**, 1141–1142.

[bb10] Sheldrick, G. M. (2008). *Acta Cryst.* A**64**, 112–122. 10.1107/S0108767307043930 18156677

[bb11] Spek, A. L. (2009). *Acta Cryst.* D**65**, 148–155. 10.1107/S090744490804362X PMC2631630 19171970

